# An Update on Osmotherapy in Acute Brain Injury: Current and Emerging Strategies

**DOI:** 10.3390/jcm15176860

**Published:** 2026-09-04

**Authors:** Sarah A. Howson, Benjamin Reddi, Rebecca J. Hood, Renée J. Turner, Mark P. Plummer

**Affiliations:** 1 Central Adelaide Local Health Network, Adelaide, SA 5000, Australiamark.plummer@sa.gov.au (M.P.P.); 2 College of Health, Adelaide University, Adelaide, SA 5000, Australia; rebecca.hood@adelaide.edu.au (R.J.H.);

**Keywords:** osmotherapy, intracranial hypertension, intracranial pressure, cerebral oedema, hypertonic saline, mannitol, neurocritical care

## Abstract

Intracranial hypertension is a major modifiable pathway of secondary brain injury after acute neurological insults. Osmotherapy is an early cornerstone of tiered intracranial pressure management. Hypertonic saline and mannitol reliably reduce intracranial pressure through osmotic reduction in brain water, with additional haemodynamic and rheological effects. However, neither agent has shown consistent improvement in survival or long-term neurological recovery. Current practice is therefore guided by physiological rather than patient-centred outcomes. This review summarises the physiological basis, clinical evidence, and practical limitations of conventional osmotherapy, and explores investigational chloride-free osmotherapies including sodium lactate, sodium bicarbonate, sodium ascorbate, and sodium acetate. Chloride-free therapies aim to preserve sodium-driven osmotic efficacy while reducing chloride exposure or targeting metabolic and oxidative pathways implicated in secondary brain injury. At present, evidence is limited to small physiological trials, retrospective cohort studies, and mechanistic human studies. Future progress requires large collaborative trials that combine pragmatic treatment comparisons with multimodal neuromonitoring, biochemical safety endpoints and patient-centred outcomes.

## 1. Introduction

Intracranial hypertension is a final common pathway of multiple neurological diseases and may result from diffuse or focal increases in brain volume, space occupying lesions, obstruction of cerebrospinal fluid (CSF) circulation, impairment of cerebral venous drainage, or disorders of intracranial pressure regulation (Table 1). In acute brain injury (ABI), raised intracranial pressure (ICP) is a key modifiable driver of secondary brain injury and a central target of neurocritical care.

The physiological basis of the intra-cranial pressure volume relationship is described by the Monro–Kellie doctrine. The cranial vault is a fixed volume compartment containing brain parenchyma, CSF, and blood; an increase in one component must be offset by a decrease in another to maintain stable ICP. Once compensatory mechanisms are exhausted, even modest increases in intracranial volume can produce dramatic rises in ICP, with consequent impairment of cerebral perfusion, cerebral ischaemia, brain herniation and death [1].

**Figure 1:**
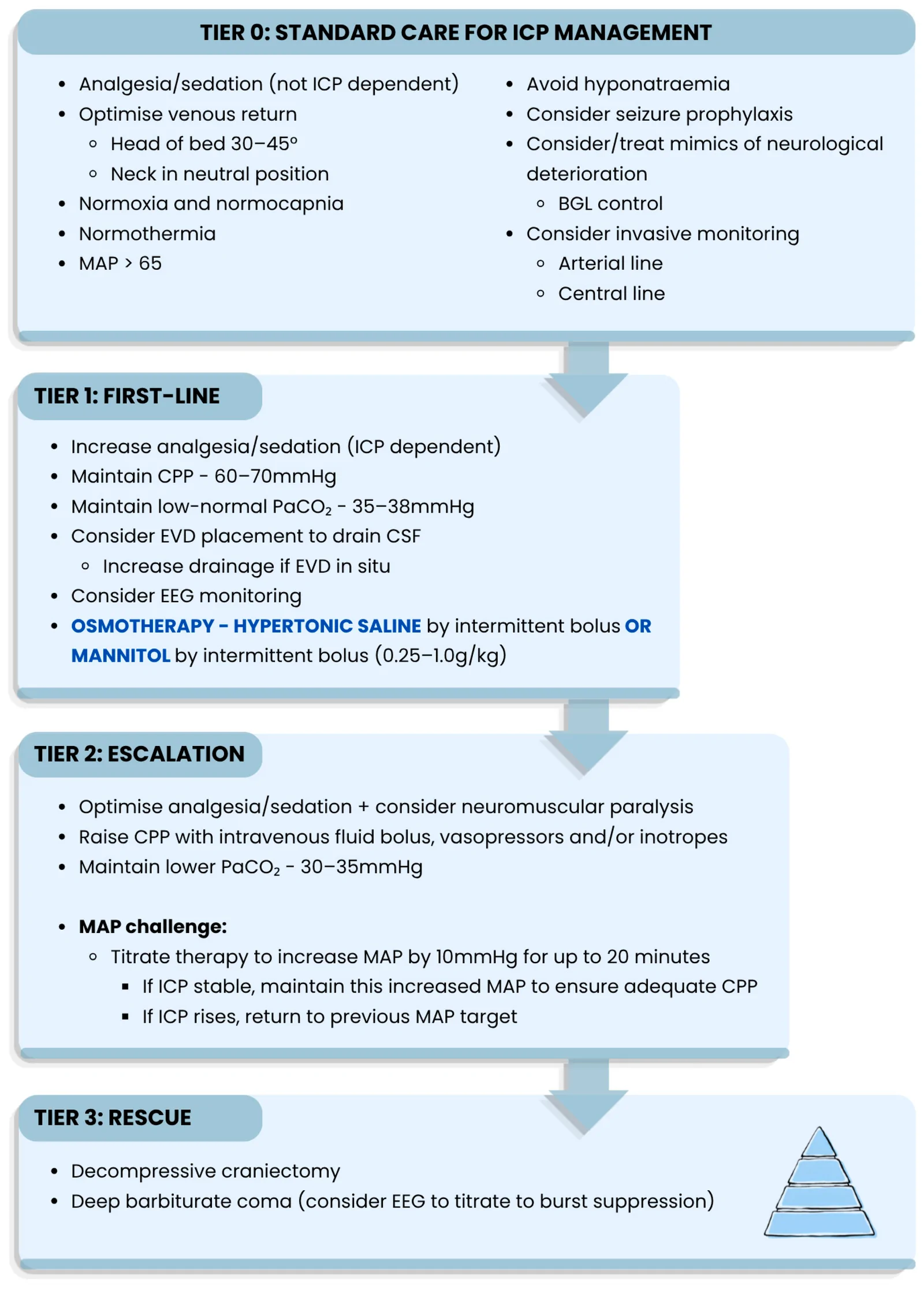
A schematic representation of the tiered management algorithm for increased ICP, adapted from Hawryluk et al. (2019) [3].

Early recognition and timely treatment of intracranial hypertension are central to neuroprotection after ABI. Contemporary management is structured around tiered escalation algorithms, which move from physiological optimisation to progressively higher-risk interventions (Figure 1) [2,3]. Osmotherapy with hypertonic saline (HTS) or mannitol is an early cornerstone of this approach as both agents are familiar, generally safe, and rapidly deployable [4].

HTS and mannitol reliably reduce ICP, yet the optimal agent, concentration, dosing strategy, and treatment target are incompletely defined. Comparative trials and meta-analyses suggest broadly similar short-term ICP-lowering efficacy, with effects typically lasting 4–6 h, with some physiological advantages favouring HTS, but neither therapy has shown consistent superiority for mortality or long-term neurological recovery [5,6,7,8]. Consequently, bedside use is largely shaped by local protocol, clinician familiarity, and patient physiology.

This review evaluates the physiological basis and clinical evidence for conventional osmotherapy with HTS and mannitol, in ABI. We also identify the limitations of traditional osmotherapies and review the rationale and emerging data for novel alternative chloride-free or biologically active sodium-based therapies, including sodium bicarbonate, sodium lactate, sodium ascorbate, and sodium acetate.

## 2. Methodology

A narrative literature review was undertaken to identify clinical, preclinical, physiological, and mechanistic evidence relating to osmotherapy for the management of cerebral oedema and raised intracranial pressure. Non-exhaustive literature searches were performed using PubMed and MEDLINE from inception to 1 May 2026. Search terms combined (including alternate spellings): traumatic brain injury, cerebral oedema, intracranial pressure, osmotherapy, mannitol, hypertonic saline, sodium lactate, sodium bicarbonate, sodium ascorbate, and sodium acetate. Reference lists of included articles and reviews were also screened to identify additional relevant studies.

Given the narrative style of the review and the heterogeneity of the available evidence, studies were selected according to their relevance to the clinical, physiological, and mechanistic aspects of osmotherapy, rather than according to formal systematic-review methodology. Peer-reviewed clinical and preclinical studies, relevant physiological and mechanistic studies, and contemporary guidelines and consensus statements were considered. For emerging therapies for which clinical evidence was limited or unavailable, preclinical and mechanistic evidence was included to contextualise proposed mechanisms of action, potential therapeutic effects, and translational potential. Papers not available in English were not screened further.

## 3. Physiological Basis of Conventional Osmotherapies

Hyperosmolar therapies lower ICP primarily by increasing plasma osmolality and establishing an osmotic gradient across the blood–brain barrier (BBB). The magnitude of this effect depends on the BBB reflection coefficient of the administered therapy. While many conditions associated with raised ICP are characterised by some degree of BBB disruption, osmotherapy remains effective provided the osmotic agent remains predominately intravascular and does not substantially accumulate within the brain parenchyma [9]. When the BBB is sufficiently intact, water moves from the intracellular and interstitial compartments of the brain into the intravascular compartment, reducing brain water content and intracranial volume, thereby lowering ICP [9]. Additional haemodynamic and rheological effects may contribute to ICP reduction by reducing erythrocyte swelling and blood viscosity, and improving cerebral microvascular perfusion with resultant autoregulatory reductions in blood volume [9].

The integrity of the BBB plays a vital role in the effectiveness of osmotherapy. Under normal physiologic conditions, the BBB restricts passage of solutes, allowing a sustained osmotic gradient to be maintained. However, in ABI, BBB disruption may permit osmotic agents to enter brain parenchyma, decreasing or reversing the osmotic gradient. With repeated or prolonged administration, particularly with mannitol, accumulation of solute within brain tissue can occur, potentially exacerbating existing cerebral oedema or contributing to rebound intracranial hypertension [10]. Rebound intracranial hypertension may precipitate secondary brain injury and necessitate escalation to higher-tier therapies.

Without clear guideline preference, agents are typically selected according to the pattern of intracranial hypertension, haemodynamic status, serum sodium, osmolality, acid–base profile, renal function and cumulative exposure to sodium, chloride, or mannitol (Table 2).

### 3.1. Hypertonic Saline

#### 3.1.1. Mechanism and Pharmacodynamics

HTS is a sodium chloride solution available in concentrations above plasma tonicity, commonly ranging from 3% to 23.4%. Its principal effect is sodium driven hyperosmolality, which draws water from brain parenchyma to the intravascular compartment and reduces ICP. Unlike mannitol, HTS expands the intravascular compartment with comparatively less osmotic diuresis, reducing the risk of hypovolaemia and haemodynamic instability [11]. Experimental data also suggest that HTS may exert an immunomodulatory effect, with reductions in leukocyte adhesion and modulation of inflammatory cascades within the cerebral microvasculature [12]. Additional preclinical studies have proposed effects on glutamate handling and excitotoxic pathways by restoring extracellular sodium and cellular action potential in the hypoxic brain, although the clinical relevance of these non-osmotic effects is uncertain [12,13].

#### 3.1.2. Clinical Efficacy

HTS reliably reduces ICP in ABI [4,5,6,14]. In traumatic brain injury (TBI), HTS is comparable to mannitol for acute ICP reduction, with some evidence of more sustained ICP control and higher CPP after treatment [5,14]. These physiological advantages may be particularly relevant in patients with extracranial injury or haemodynamic instability, where hypotension can compound secondary brain injury. However, HTS has not demonstrated superiority over mannitol for mortality or long-term neurological outcome, and the available evidence is limited by small, often single-centre randomised trials, heterogeneous dosing regimens (including variability in concentration, bolus versus continuous infusion strategies, and treatment thresholds), and inconsistent outcome definitions [5,7,10,11,15].

#### 3.1.3. Limitations and Considerations

HTS requires close physiological and biochemical monitoring. Hypernatraemia is an expected pharmacological effect, but rapid changes in serum sodium may be harmful, particularly in patients who are relatively hyponatraemic at baseline. Serum sodium targets of 145 to 155 mmol/L are commonly used in protocols and clinical studies, although the Brain Trauma Foundation does not recommend a specific sodium target [2]. HTS can promote diuresis, both through plasma volume expansion and osmotic diuresis; this may precipitate hypokalaemia, necessitating regular electrolyte monitoring and replacement.

Significant HTS administration may be associated with a potentially injurious chloride load. For example, 3% sodium chloride contains approximately 513 mmol/L of each ion. Systemically, excess chloride reduces the strong ion difference and promotes a normal anion gap metabolic acidosis [16]. In critically ill patients, acidaemia may impair myocardial contractility, reduce vascular responsiveness to catecholamines, increase respiratory drive and complicate ventilatory control in patients where arterial carbon dioxide is tightly managed [15,17]. Hyperchloraemia has been linked to renal vasoconstriction, reduced renal blood flow and acute kidney injury (AKI) in broader critical care cohorts, although the relationship between chloride exposure and renal injury in ABI appears less consistent [18,19].

Observational data consistently associate higher chloride with worse outcomes. In severe TBI, recurrent early hyperchloraemia has been associated with increased 180-day mortality, and time-weighted chloride exposure has been associated with early mortality independent of concomitant hypernatraemia [20,21]. However, hyperchloraemia may also reflect illness severity, resuscitation intensity, and greater exposure to hyperosmolar therapy. Importantly, these observational associations do not establish that a reduction in chloride exposure would improve clinical outcomes. The current evidence therefore supports active monitoring of serum chloride and acid–base status during prolonged HTS therapy but does not establish a chloride threshold at which treatment should be withheld [2].

HTS expands intravascular volume which may be advantageous in hypovolaemic patients. However, repeated dosing or continuous infusion can contribute to fluid overload and pulmonary oedema in susceptible patients [9]. Careful assessment of volume state is therefore required during prolonged therapy.

Finally, practical considerations include the requirement for reliable venous access. Although peripheral administration of certain hypertonic solutions is feasible, higher concentrations of HTS can require central venous access to reduce the risk of local irritation and extravasation injury, in accordance with institutional protocol [9]. This may influence agent selection in time-critical settings where central access is not immediately available.

#### 3.1.4. Intermittent Bolus vs. Continuous Dosing

HTS may be administered as intermittent boluses or as a continuous infusion. Bolus therapy treats acute ICP elevation and permits titration against a defined physiological response. This allows repeat dosing or escalation when needed, while avoiding ongoing sodium and chloride exposure once ICP is controlled. Continuous infusion is usually titrated to a predefined serum sodium or osmolality range. The aim is to reduce ICP variability and prevent recurrent intracranial hypertension, particularly in patients with sustained or refractory ICP elevation. The trade-off is greater cumulative exposure to sodium and chloride, requiring close monitoring of sodium, chloride, osmolality, acid-base status, renal function, and fluid balance.

Evidence comparing intermittent bolus or continuous infusion strategies is limited. The largest randomised trial evaluated early continuous infusion of 20% hypertonic saline in 370 adults with moderate to severe TBI across nine French intensive care units [22]. Patients were randomised within 24 h of injury to continuous 20% hypertonic saline plus standard care, or standard care alone. The infusion was continued for at least 48 h, or longer if patients were considered at ongoing risk of intracranial hypertension, with dosing adjusted against serial serum sodium measurements. Continuous therapy increased serum sodium and osmolality and may have improved early ICP control, but this did not translate into demonstrable functional benefit, with no improvement in neurological outcomes or mortality at six months [22]. Current evidence does not establish superiority of continuous infusion over intermittent bolus therapy.

An important consideration during prolonged hypertonic therapy, and a key challenge for the concept of prophylactic osmotherapy, is cerebral osmotic adaptation [10]. In response to sustained extracellular hypertonicity, astrocytes and other brain cells accumulate intracellular organic osmolytes, including taurine, myo-inositol, betaine and glutamine [23]. These “idiogenic osmoles” restore intracellular osmolality and cell volume despite persistent hypernatraemia [10]. While this adaptation protects against excessive cellular dehydration, it may progressively reduce the effectiveness of a fixed osmotic gradient over time. Furthermore, rapid reduction or cessation of prolonged hypertonic therapy may create a reverse osmotic gradient, promoting cerebral water accumulation and rebound intracranial hypertension [10]. These physiological considerations provide a mechanistic rationale for favouring intermittent treatment of ICP crises, targeted to ICP itself, over routine prophylactic hypernatraemia, targeting serum sodium concentration, although the optimal administration strategy remains uncertain.

### 3.2. Mannitol

#### 3.2.1. Mechanism and Pharmacodynamics

Mannitol is a water-soluble sugar alcohol that acts as an osmotic diuretic. Following intravenous administration, it increases plasma osmolality and establishes an osmotic gradient across the BBB, drawing water from brain tissue into the intravascular compartment. This reduces brain water content, intracranial volume and ICP, provided that the BBB remains sufficiently intact to maintain the osmotic gradient [9].

#### 3.2.2. Clinical Efficacy

Mannitol is an established ICP-lowering therapy, with extensive data supporting short-term efficacy in the setting of traumatic and non-traumatic intracranial hypertension [4,24]. In addition to its osmotic effect, it may also confer rheological benefits through reduction in blood viscosity and improvements in cerebral microcirculatory flow. However, as with HTS, these physiological benefits have not translated into consistent evidence of improved mortality or long-term functional outcome [7,10].

#### 3.2.3. Limitations and Considerations

The major limitation of mannitol is its dynamic, profound effect on circulating blood volume. Mannitol draws water from the intracellular to extracellular compartment, causing transient intravascular volume expansion. While this may temporarily augment cardiac output and blood pressure, it can precipitate pulmonary congestion or worsen heart failure in susceptible patients [24]. Mannitol is freely filtered by the glomerulus and can precipitate osmotic diuresis leading to hypovolaemia, hypotension and reduced CPP, particularly in patients with shock, renal dysfunction or limited physiological reserve [24].

Electrolyte disturbance is also common. Hyponatraemia can occur when mannitol shifts water from the intracellular to extracellular compartment, followed by hypernatraemia when free water excretion during osmotic diuresis exceeds sodium excretion. Hypokalaemia may occur as mannitol also augments distal sodium and water delivery promoting urinary potassium loss [24]. Serum osmolality is frequently used to guide repeated mannitol dosing; a threshold of 320 mOsm/kg has traditionally been used as a pragmatic upper limit [15]. Osmolar gap may better reflect circulating mannitol concentration than serum osmolality alone when monitoring for accumulation, although this recommendation is based on very low-quality evidence [15]. In practice, redosing should not be guided by a single osmolality or osmolar gap value in isolation, but by integrated assessment of ICP response, mean arterial pressure (MAP) and CPP, volume status, renal function, electrolyte trends, and cumulative dose.

This concern regarding accumulation is not limited to the intravascular compartment. In conditions associated with more extensive BBB disruption, mannitol may enter injured brain tissue and reduce or even reverse the osmotic gradient across the BBB. With large or repeated doses, particularly in the setting of severe BBB disruption or impaired renal clearance of mannitol, this may contribute to reduced efficacy or rebound intracranial hypertension after cessation [24]. This risk is difficult to quantify clinically but mandates caution with repeat dosing in patients with extensive brain injury or renal dysfunction [25].

Mannitol can also contribute to AKI through both haemodynamic and direct tubular mechanisms. Because mannitol is freely filtered but not reabsorbed, high cumulative doses may exceed renal excretory capacity, leading to intrarenal accumulation and osmotic nephrosis, characterised by swelling of proximal tubular cells [24]. In parallel, the osmotic diuresis produced by mannitol may cause hypovolaemia, reducing renal perfusion and predisposing to acute tubular injury [24]. The risk of AKI appears to increase when mannitol dosing is high or when haemodynamic compromise persists [24].

#### 3.2.4. Intermittent Bolus vs. Continuous Dosing

Mannitol is administered almost exclusively as intermittent bolus therapy. Standard doses range from 0.25 to 1 g/kg, usually infused over 5 to 20 min. Bolus dosing allows reassessment of ICP, CPP, volume status and renal function before further treatment. Continuous infusion is generally avoided because it increases the risk of accumulation, progressive hyperosmolality, renal toxicity and rebound intracranial hypertension [24].

## 4. Investigational Hyperosmolar Therapies

Although mannitol and HTS reliably reduce ICP, both are conventional osmotic therapies that primarily target intracranial volume rather than the broader mechanisms of secondary brain injury. Their use is also limited by treatment-specific toxicity, including hypovolaemia, renal dysfunction and rebound intracranial hypertension with mannitol, and hyperchloraemic metabolic acidosis with hypertonic saline. These limitations have prompted growing interest in two related strategies: chloride-free osmotherapies that aim to avoid possible treatment-related harm, and biologically active agents that may combine ICP reduction with effects on secondary brain injury pathways [26,27,28,29].

Chloride-free hyperosmolar fluids that may also provide additional neuroprotective effects are attractive candidate therapies. Sodium bicarbonate and sodium acetate are principally chloride-free strategies (Table 3). Sodium lactate and sodium ascorbate provide a potential metabolic or antioxidant benefit (Table 3). At present, these therapies should be considered investigational rather than established replacements for HTS or mannitol.

For these investigational agents, available evidence is predominately limited to physiological and biochemical endpoints, with no adequately powered evidence demonstrating improved neurological or patient-centred outcomes (Table 4).

### 4.1. Sodium Lactate

Sodium lactate provides a chloride-free hyperosmolar sodium load and may also confer metabolic benefits within the injured brain. Lactate is an important intermediary in cerebral energy metabolism and is transported across the BBB through monocarboxylate transporters [30]. Within the brain, lactate can be used by neurons and astrocytes to support mitochondrial ATP generation, a potential advantage in ABI where impaired glucose utilisation, mitochondrial dysfunction, and cellular energy failure contribute to secondary brain injury [30,31,32]. Sodium lactate therefore has a plausible therapeutic rationale that extends beyond osmotic ICP reduction.

The evidence for sodium lactate is more developed than for most chloride-free alternatives but remains confined to physiological endpoints. In a double-blind randomised trial, 60 patients with severe TBI requiring ICP monitoring were allocated within 12 h of injury to a 48 h continuous infusion of half-molar sodium lactate or isotonic saline, both administered at 0.5 mL/kg/h [33]. Sodium lactate reduced intracranial hypertensive episodes and was associated with lower cumulative fluid and chloride balances [33]. Bernini and colleagues subsequently reported a single-centre retrospective within-patient cohort study of 17 patients with severe ABI, including 13 with TBI, who received sequential equiosmolar and isovolumic boluses of 7.5% hypertonic saline and hypertonic lactate for sustained ICP elevation [26]. Both agents produced comparable ICP reductions in clinically meaningful magnitude, lasting 2.5–3 h. Hypertonic lactate avoided the chloride rise and hyperchloraemic acidosis observed with hypertonic saline [26]. Neither treatment produced consistent improvement in brain tissue oxygenation or cerebral microdialysis markers [26]. Although these data support sodium lactate as a biologically plausible chloride-free osmotherapy with a favourable biochemical profile, data regarding patient outcomes are absent and are insufficient to support routine use over established therapies.

### 4.2. Sodium Bicarbonate

Sodium bicarbonate is a chloride-free hyperosmolar sodium solution. The most widely available formulation, 8.4% sodium bicarbonate, contains approximately 1 mmol/mL of sodium and bicarbonate and has an osmolarity of approximately 2000 mOsm/L. It therefore delivers a substantial sodium and osmotic load without chloride, making it conceptually attractive when ongoing osmotherapy is complicated by hyperchloraemia or normal anion gap metabolic acidosis.

Clinical evidence for sodium bicarbonate osmotherapy (SBO) is limited but supports a short-term ICP-lowering effect. In an uncontrolled prospective pilot study, seven patients with severe TBI received 85 mL of 8.4% sodium bicarbonate for raised ICP, with rapid ICP reduction sustained over 6 h and no associated hyperchloraemia [34]. A subsequent small, open-label randomised trial enrolled 11 patients with severe TBI and compared equiosmolar 8.4% sodium bicarbonate with 5% sodium chloride across 20 episodes of intracranial hypertension [27]. Both agents reduced ICP, with no significant difference in ICP response over time [27]. SBO increased arterial pH, while sodium chloride produced greater chloride exposure [27]. Clinical outcome data were not recorded.

Whilst the available data suggest that SBO can produce clinically relevant reductions in ICP, the lack of comparative data indicating benefit has prevented SBO entering standard neurocritical care practice. Potential adverse effects of SBO include metabolic alkalosis, hypernatraemia, hyperosmolality, hypocalcaemia, and carbon dioxide generation. The latter is relevant in ABI because carbon dioxide crosses the BBB rapidly and may influence cerebral blood flow if ventilation is not tightly controlled. Effects of SBO on brain tissue oxygenation, cerebral metabolism, and long-term neurological outcome are unknown. Sodium bicarbonate should therefore be regarded as a promising chloride-free osmotherapy with early signals of physiological efficacy that requires further validation in clinical trials to assess clinical benefit.

### 4.3. Sodium Ascorbate

Sodium ascorbate is a pH-neutral, chloride-free sodium salt of vitamin C that can be prepared as a hyperosmolar solution for ICP reduction [29], reducing brain water by increasing plasma osmolality and drawing water from brain tissue into the intravascular compartment. Concurrently, sodium ascorbate delivers pharmacological concentrations of ascorbate, an important endogenous neuronal antioxidant, which may have neuroprotective properties [35]. The brain is highly susceptible to oxidative injury due to its high oxygen consumption and abundant polyunsaturated fat content [29]. ABI is characterised by oxidative stress, mitochondrial dysfunction, BBB disruption and neuroinflammation. Following ABI, plasma and CSF ascorbate concentrations fall rapidly, with lower concentrations associated with greater brain tissue injury burden [36,37]. As such, depletion of endogenous ascorbate may represent both a marker of injury burden and a potential therapeutic target, although whether restoration of ascorbate concentrations improves patient-centred outcomes remains unknown.

Clinical experience with ascorbate-containing osmotherapy is very limited with no standalone human trials. Oppido and colleagues reported an uncontrolled case series of 80 patients with brain oedema or intracranial hypertension treated with GLIAS, a buffered hyperosmolar solution containing 30% glycerol, 20% sodium ascorbate, and 0.2% tioglycerol [38]. Participants suffered post-traumatic, peri-tumoural, post-operative and intra-operative cerebral oedema. The authors reported increased plasma osmolarity, reduced ICP, improved cerebral compliance and no major disturbance in fluid or electrolyte balance. However, an effect of sodium ascorbate cannot be distinguished from the confounding effects of glycerol (an established osmotic agent) and tioglycerol.

There are currently no published clinical trials evaluating sodium ascorbate alone for ICP reduction in ABI. Key uncertainties include dose, duration of effect, adverse consequences of oxalate generation in patients with impaired clearance, interference with glucose and ketone assays, and safety in patients with glucose-6-phosphate dehydrogenase deficiency [29]. A phase 1 clinical study evaluating 30% sodium ascorbate in adults with moderate to severe TBI requiring invasive neuromonitoring and treatment for raised ICP has been registered, and clinical safety, pharmacokinetic and pharmacodynamic data are awaited (ACTRN12625000178448). Sodium ascorbate should therefore be regarded as an investigational chloride-free osmotherapy with a plausible mechanistic rationale, requiring evaluation as a standalone intervention.

### 4.4. Sodium Acetate

Sodium acetate is best understood as a chloride-free modification of established hypertonic sodium therapy. In contrast to lactate and ascorbate, its rationale is not primarily based on direct cerebral metabolic or antioxidant effects. Rather, acetate allows preservation of hypertonic sodium delivery while reducing chloride exposure and providing a metabolised anion that can generate bicarbonate.

Acetate is metabolised predominantly in peripheral tissues to bicarbonate, which avoids the hyperchloraemic metabolic acidosis associated with HTS therapy. This makes acetate-containing formulations particularly useful when ongoing osmotherapy is required in the setting of rising chloride, worsening base deficit, or concern regarding renal injury.

Clinical evidence is limited but supports physiological efficacy. In a retrospective series of 27 neurocritical care patients, continuous, variable rate 3% saline/acetate was used to achieve a serum sodium concentration of 145 to 155 mmol/L, or until complications arose [39]. Included patients had cerebral oedema due to head trauma, post-operative oedema, non-traumatic intracranial haemorrhage, or cerebral infarction [39]. ICP decreased within 12 h in patients with head trauma and post-operative oedema, which correlated with an increase in serum sodium, supporting a sodium-driven osmotic effect [39]. However, the effect in head trauma appeared short-lived and the response was less evident in non-traumatic haemorrhage and infarction [39]. In a subsequent single-centre, double-blind, double-dummy randomised trial of 32 patients with aneurysmal subarachnoid haemorrhage, a lower-chloride sodium chloride/sodium acetate formulation was compared with 23.4% sodium chloride in patients with hyperchloraemia requiring further hyperosmolar therapy [28]. The acetate-containing formulation produced similar ICP reduction, delivered a lower chloride load, and was associated with a lower rate of AKI [28]. However, the trial was small, restricted to subarachnoid haemorrhage, and not powered for patient-centred outcomes.

In a single-centre chart review of patients receiving hypertonic therapy for ICP management, acetate-buffered 3% hypertonic saline produced a similar increase in serum osmolality to standard 3% hypertonic saline over 72 h [40]. In patients switched from standard 3% hypertonic saline to the acetate-buffered formulation, serum chloride decreased, while matched patients continuing standard 3% hypertonic saline had rising chloride concentrations [40].

Together, these data support sodium acetate as a pragmatic chloride-free modification of conventional HTS practice, but comparative effectiveness, optimal formulation, vascular tolerability, acid–base effects and effects on renal or neurological outcomes remain uncertain. Larger comparative trials are required before acetate-containing solutions can be recommended as routine alternatives to conventional HTS.

**Table 4:** Summary of investigational osmotherapy studies.

Agent	Authors	Design	Sample Size	Intervention/Comparator	Primary/Reported Endpoint
Sodium lactate	Ichai et al. (2009) [32]	Prospective open-label randomised	34 patients	Sodium lactate 0.5 M vs. mannitol 20%	ICP response
Sodium lactate	Ichai et al. (2013) [33]	RCT	60 patients	Sodium lactate 0.5 M vs. isotonic saline 0.9%	Intracranial hypertensive episodes
Sodium lactate	Bernini et al. (2022) [26]	Retrospective within-patient cohort study	17 patients; 34 episodes	Sodium lactate equiosmolar/isovolumic vs. hypertonic saline 7.5%	ICP response
Sodium bicarbonate	Bourdeaux & Brown (2010) [34]	Prospective pilot	7 patients; 10 episodes	Sodium bicarbonate 8.4%	ICP response
Sodium bicarbonate	Bourdeaux & Brown (2011) [27]	RCT	11 patients; 20 episodes	Sodium bicarbonate 8.4% vs. sodium chloride 5%	ICP response
Sodium ascorbate ^1^	Oppido et al. (1992) [38]	Case series	80 patients	Glycerol 30% + sodium ascorbate 20% (GLIAS)	ICP response; plasma/urine osmolarity
Sodium acetate ^2^	Qureshi et al. (1998) [39]	Retrospective chart review	27 patients	Hypertonic saline 3% with sodium acetate buffer	ICP response
Sodium acetate	Sadan et al. (2020) [28]	Randomised pilot	32 patients	Sodium chloride/sodium acetate 16.4% vs. hypertonic saline 23.4%	Serum chloride level
Sodium acetate ^2^	Holden et al. (2021) [40]	Retrospective chart review	44 patients	Hypertonic saline 3% vs. hypertonic saline 3% with sodium acetate buffer	Serum osmolality; chloride exposure

^1^ GLIAS is a combination preparation containing Glycerol 30% + sodium ascorbate 20%; efficacy attributable specifically to sodium ascorbate cannot be determined by this study. ^2^ Authors use sodium acetate as a component/buffer of hypertonic saline; efficacy attributable specifically to sodium acetate cannot be determined by this study.

## 5. Future Directions

Despite decades of research and near-universal clinical use, there is still no high-quality evidence that osmotherapy improves neurological outcome after ABI. This evidence gap is reflected in the Brain Trauma Foundation guidelines, which do not identify a preferred osmotic agent, concentration, dosing strategy, or biochemical target despite osmotherapy being embedded in routine care [2]. Observational comparative-effectiveness data further highlight the difficulty of interpreting osmotherapy choice in routine practice, where treatment selection is strongly shaped by illness severity, physiology, and clinician preference [41].

Addressing this evidence gap requires large, pragmatic trials that reflect bedside practice. Future trials should also determine how treatment should be initiated, including whether osmotherapy is best guided by absolute ICP thresholds or cumulative ICP burden, individualised haemodynamic parameters, or specific metabolic targets. Integration with multimodal neuromonitoring and metabolic measures may allow treatment to be better matched to the underlying physiological disturbance. For metabolically active osmotherapy it is important to distinguish and optimally harness osmotic versus metabolic and antioxidant effects.

Safety monitoring should extend beyond serum sodium and osmolality to include chloride exposure, acid–base status, osmolar gap, renal function, fluid balance, cumulative osmotherapy dose, and other treatment toxicities. While a rationale for chloride-free agents is acknowledged, it is yet to be shown whether they can reduce ICP more safely than existing therapies and with demonstrable clinical benefit.

Outcome studies should relate treatment targets, agent selection, and administration strategies to ICP burden, broader physiological outcomes and, most importantly, long-term functional neurological outcomes, with consideration of quality of life, early and late functional outcome, and degree of disability measured by validated scales such as the Glasgow Outcome Scale-Extended or the Functional Independence Measure [42].

## 6. Conclusions

Osmotherapy is a core component of tiered intracranial pressure management and reliably reduces intracranial pressure. However, evidence of clinical benefit is limited, and treatment-related toxicity is increasingly relevant when osmotherapy is repeated or prolonged. Chloride-free and biologically active osmotherapies offer a rational next step but require rigorous clinical evaluation. Future trials must determine whether conventional and investigational osmotherapy can translate physiological efficacy into improved patient-centred outcomes.

## Figures and Tables

**Table 1:** Intracranial causes of intracranial hypertension.

Pathophysiology	Cause
Diffuse brain oedema	Encephalopathies (toxic, uraemic, septic, hepatic, hypoxaemic ischaemic), encephalitis, meningitis, traumatic brain injury
Space occupying lesion	Haematoma (extradural, subdural, intracerebral), abscess, neoplasm
Focal brain oedema	Associated with space occupying lesions, ischaemic stroke, subarachnoid haemorrhage
Impedance of CSF flow	Obstructive or communicative hydrocephalus, aqueductal stenosis, choroid plexus papilloma
Impedance of blood flow	Venous sinus thrombosis, jugular vein compression
Congenital malformation	Dandy-Walker malformation, Chiari malformation, arteriovenous malformation
Idiopathic	Idiopathic intracranial hypertension

**Table 2:** Direct comparison between HTS and Mannitol.

	Hypertonic Saline	Mannitol
Primary mechanism	Sodium driven hyperosmolality	Osmotic diuretic increases plasma osmolality and renal free water excretion
Reflection coefficient	~1.0 (largely excluded from intact BBB ^1^)	~0.9 (partial BBB ^1^ permeability)
Intravascular volume effect	Expands intravascular volume via osmotic fluid shift	Initial expansion followed by diuresis and potential hypovolaemia
Cerebral perfusion pressure effect	Increases or maintains CPP ^2^ via volume expansion and MAP ^3^ support	May decrease CPP ^2^ if hypovolaemia develops
Secondary systemic effects	Hypernatraemia, hyperchloraemia, metabolic acidosis, hypervolaemia	Hypovolaemia, electrolyte disturbance, risk of AKI ^4^, risk of rebound ICP ^5^ increase
Administration considerations	Can require central venous access for higher concentrations (e.g., 23.4%) in accordance with institutional protocol and duration of administration; bolus dosing or continuous infusion	Peripheral venous access; bolus dosing

^1^ BBB—Blood–brain barrier; ^2^ CPP—Cerebral perfusion pressure; ^3^ MAP—Mean arterial pressure; ^4^ AKI—Acute kidney injury; ^5^ ICP—Intracranial pressure.

**Table 3:** Summary of investigational osmotherapies.

Agent	Osmotic Effect	Secondary Effect	Key Advantage	Major Limitation
Sodium lactate	Yes	Alternative cerebral energy source	Chloride-free	No comparative outcome benefit shown
Sodium bicarbonate	Yes	Alkalinisation	Chloride-free	No comparative outcome benefit shown, alkalosis, hypocalcaemia, CO_2_ generation
Sodium ascorbate	Yes	Antioxidant, directly scavenges free radicals	Neuroprotective rationale; chloride-free	No standalone human trial
Sodium acetate	Yes	Alkalinisation via HCO_3_^−^ generation from acetate	Chloride-free	No comparative outcome benefit shown

## Data Availability

No new data were created or analysed in this study. Data sharing is not applicable to this article.

## Abbreviations

The following abbreviations are used in this manuscript:

ABI	Acute brain injury
AKI	Acute kidney injury
BBB	Blood–brain barrier
CBF	Cerebral blood flow
CPP	Cerebral perfusion pressure
CSF	Cerebrospinal fluid
HTS	Hypertonic saline
ICP	Intracranial pressure
MAP	Mean arterial pressure
ROS	Reactive oxygen species
TBI	Traumatic brain injury
